# Methods for Cryopreservation of Guinea Fowl Sperm

**DOI:** 10.1371/journal.pone.0062759

**Published:** 2013-04-29

**Authors:** Éva Váradi, Barbara Végi, Krisztina Liptói, Judit Barna

**Affiliations:** Institute for Small Animal Research and Co-ordination Centre for Gene Conservation, Gödöllő, Hungary; Clermont-Ferrand Univ., France

## Abstract

Conservation of indigenous poultry species is an important part of the new Hungarian agricultural strategy. Semen cryopreservation is the most practical method for the long term storage of poultry genetic material. The objective was to compare four protocols for cryopreservation of guinea fowl sperm (slow and fast programmable, freezing in nitrogen vapor, and pellet) and three cryoprotectants (10% ethylene glycol, 6% dimethyl-formamide and 6% dimethyl-acetamide). The efficiency of the methods was examined by *in vitro* tests (subjective motility scoring, sperm concentration, morphological and live/dead sperm analysis with eosin-aniline staining). Thereafter, the two most promising methods were tested by artificial insemination of frozen-thawed semen (3 times a week for 3 weeks using 300 million spermatozoa/hen), followed by candling of incubated eggs, assessment of fertilization, embryonic death, and hatching rate. The survival rate of live, intact spermatozoa was greatest (p≤0.05) in pellet method and the slow programmable protocol (with 10% ethylene glycol) (28.6 and 23.5%). The two best protocols (based on *in vitro* assessment of post-thaw semen quality) were subsequently tested *in vivo* with artificial insemination. The pellet method yielded a 64% fertility rate compared to slow protocol with only 30% fertility. Regardless, both freezing protocols significantly increased embryonic deaths compared to the control group (16,7; 9,1 and 8,3%, respectively). During the 3-week *in vivo* trial, fertility increased and early embryonic death decreased over time. According to the results the guinea fowl sperm could tolerate the fast freezing in pellet better than the slower freezing rates and resulted acceptable fertility rate.

## Introduction

Biodiversity and gene conservation are of broad interest all over the world. Although much of the focus is on endangered species, these issues are also important for domestic livestock. With intense animal breeding and better, more homogenous environmental conditions, genetic diversity of domestic species has decreased rapidly [1]. Intensive poultry breeding pushed the indigenous breeds into the background disposing of valuable genes. According to the DAD-IS (Domestic Animal Diversity Information System), more than 50% of poultry species are in the endangered category [2]. Consequently, there was an urgent need to create gene- and databanks [3]. It is well known that for safe gene conservation, both *in vivo* and *in vitro* gene banks are necessary. Presently, indigenous poultry genetic materials are stored *in vitro* in only four national gene banks (France, Netherlands, North-America and Spain) [4], [5], [6]. In addition to *in vivo* management, i*n vitro* conservation is a strategic tool to secure genetic diversity, considering the risk of epidemic diseases [7].

Semen cryopreservation is presently the most practical method for long-term storage of genetic material in poultry species [8], [9], [10]. The efficiency of sperm cryopreservation is variable among poultry species [11]. The freezing tolerance of guinea fowl spermatozoa seems to be the lowest among the various poultry species, presumably due to its low membrane fluidity, which was attributed to low concentrations of cholesterol. Moreover, the quality of guinea fowl semen is also lower than that most other poultry species [10].

In the apparently sole study of cryopreservation of guinea fowl spermatozoa, an intermediate freezing rate (15°C/min) and 6% dimethyl-formamide (DMF) was used; 37% sperm survival and 20% fertility were achieved [12]. *Schramm and Hübner* [13] successfully cryopreserved duck spermatozoa using a slow freezing protocol with ethylene glycol (EG). Freezing spermatozoa in nitrogen vapor has been widely used for fish [14] as well as for geese [15].

Sperm cryopreservation using slower freezing methods and pellet form using dimethyl-acetamide (DMA) looks also promising. This simple cryoconservation technique does not require complicated equipment, making it appropriate for widespread applications [16]. These methods have achieved high fertility rates for various poultry species [17], [18], although this approach has apparently not been reported for guinea fowl.

In the present study four freezing protocols with various cooling rates (slow and fast programmable method, freezing in nitrogen vapor, pellet method) and cryoprotectants (EG, DMF, DMA) were tested for cryopreservation of guinea fowl spermatozoa.

## Materials and Methods

### Ethics Statement

Keeping of animals and animal welfare prescriptions were according to the Hungarian Animal Protection Law (1998. XXVIII). Institute for Small Animal Research and Co-ordination Centre for Gene Conservation provides a permission for experimental animal research from the National Food Chain Safety Office, Animal Health and Animal Welfare Directorate, Budapest (permission number: 1/1512/49/15/2/2012). All applied experimental methods were permitted the own Institutional Ethical Review Board (No.7/2011) of the Institute for Small Animal Research and Co-ordination Centre for Gene Conservation.

### Animals

Thirty rural Hungarian guinea fowl males (1 year old) were kept in individual cages, exposed to a 16L:8D photoperiod (natural and artificial light) and given *ad libitum* access to a commercial poultry ration and to water.

### Semen Collection and Qualification

Males were selected firstly according to their tractability and response of sperm collection. Following a training period, assessment of semen quality was done to selecting the best sperm donors. For a two-month interval, semen was collected twice a week by dorso-abdominal massage [19]. During the sperm freezing period, *in vitro* assessment of semen quality was done before freezing and after thawing (fresh and frozen/thawed semen). Following sperm volume measurement, motility was determined by subjective scoring (0–5) by a well-trained person, while sperm concentration was estimated with a spectrophotometer (Accucell IMV Technologies, L’Aigle, France). Sperm morphology normal, abnormal and live/dead cell ratio was examined with aniline blue-eosin staining. This procedure is based on eosin (Certistain, 115935 Eosin Y, Merck Ltd., Budapest, Hungary) that is excluded by live, intact cell membranes but dead sperm membranes permit the eosin to stain the sperm. The aniline blue (415049 Sigma-Aldrich Ltd., Budapest, Hungary) provides a clear blue background in the smear to enhance the contrast of the white, unstained “live” sperm or the pinkish, stained “dead” sperm. Slides were evaluated microscopically (Zeiss, Axioscope; Carl Zeiss Microscopy GmbH., Göttingen, Germany) using an oil immersion objective and 1200× magnification. A total of 200 spermatozoa were assessed per slide.

### Freezing Protocols

In the study, four freezing protocols with various cooling rates (slow and fast programmable method, freezing in nitrogen vapor, pellet method) and three cryoprotectants (CPs) were tested: 10% ethylene glycol (EG; 09660-1-01-65, Reanal Laborvegyszer Ldt. Budapest, Hungary), 6% dimethyl-formamide (DMF; 444926, Carlo Erba Reagenti, Arese, Italy) and 6% dimethyl-acetamide (DMA; Sigma-Aldrich Ltd, Budapest, Hungary) *(* *Table 1* *).*

**Table 1:** Experimental design of freezing protocols.

Protocols	Slow programmable		Fast programmable		Nitrogen vapor		Pellet
**Type of cryo container**	Cryo vial						
**Diluents**	Lake’s						Tselutin’s
**Dilution rate**	1∶3						1∶1
**Equilibration time**	25 min at 3°C		5 min at 5°C		5 min at 5°C		5 min at 2°C
**CPs**	10%EG	6%DMF	10%EG	6%DMF	10%EG	6%DMF	6%DMA
**Freezing rate**	−1°C/min to −30°C		−15°C/min to −30°C		4 cm above LN		directly into
	−30°C/min to −60°C		−30°C/min to −60°C		(120°C for 3 min)		LN

### Slow Programmable Protocol

After sperm collection, pooled semen was diluted 1∶3 with Lake’s extender [20] at room temperature. Diluted semen was divided in two equal parts: one sample contained 10% EG, the other 6% DMF as cryoprotectants. Aliquots (200 µL) of the samples were measured into cryo vials and placed into a programmable freezing machine (Planer KRYO10, Planer Products Ltd., Middlesex, U.K). Cooling and freezing was carried out in three consecutive steps, as described [12] with some modifications: Cooling was started at 20°C, with a −3°C/min freezing rate until 3°C, followed by 25 min equilibration. Then, a −1°C/min freezing rate was applied from 3°C to −30°C, than −30°C/min rate to −60°C. Finally cryo vials were placed into liquid nitrogen (LN). Samples were frozen for at least 2 weeks. Thawing was carried out at 5°C for 30 min in a cooler cabin (Brillant EURO-1-E, Ausseer Kälte und Edelstahltechnik GmbH, Kainisch, Austria).

### Fast Programmable Protocol

Pretreatment of the semen was similar to the previous protocol, except the equilibration period, which lasted 5 minutes at 5°C for both 10% EG or 6% DMF. Aliquots (200 µL) from the samples were put into the cryo vials and placed in the freezing machine. Freezing started at 5°C with an intermediate freezing rate (−15°C/min) until −30°C, than samples were cooled (−30°C/min freezing rate) to −60°C. Cryo vials were placed into liquid nitrogen (LN). Thawing was done out at 5°C for 20 min in a cooler cabin (as described above).

### Freezing in Nitrogen Vapor

Dilution and precooling of the semen were exactly the same as in fast programmable protocol. However, the cryo vials were put onto a vial rack which was placed in a plastic box at 4 cm above the liquid nitrogen. Thawing was carried out at 38°C in an incubator for 3 min (NB-201Q, N-Biotek Inc., Korea).

### Pellet Method

After sperm collection, pooled semen was diluted 1∶1 with Tselutin’s extender [17] at room temperature. Cooling was 20 min at 2°C, whereas in the last 2 minutes 6% DMA was added to the sample. Treated semen (25 µL) was directly dropped into the liquid nitrogen using a mechanical pipette (Eppendorf Multipette plus, Eppendorf Austria GmbH, Wien, Austria). Pellets were collected with forceps and put in cryo vials filled with LN, which were then stored in a container containing LN. Thus, inside the vials the pellets were also in LN. Thawing was done at 70°C for 10 sec in a self-fabricated automatic warming device. The content of the vials was poured into the preheated metal funnel of the device and the thawed sperm was collected into a glass vessel.

### Artificial Insemination

Thirty female guinea fowls were placed in individual cages (conditions were similar to those used for the males). The two best freezing methods (based on *in vitro* assessment of frozen-thawed sperm) were tested with artificial insemination (as an *in vivo* qualification method). Ten females (controls) were inseminated with fresh, diluted semen, whereas the remaining 20 were inseminated with frozen/thawed semen from slow and pellet method (10 birds per protocol). Artificial insemination was done within 10 minutes after thawing thrice weekly for 3 weeks using 250–300 million spermatozoa/hen/AI. The fertility of 300 incubated eggs was checked by candling at the 10^th^ day of incubation. The rate of the true fertility (normal and dead embryos) was determined. Embryos that had died in the oviduct were detected by propidium iodide staining of the germinal disc. Egg yolks were separated from the albumen and placed into 0.9% NaCl solution. After visual fertility examination germinal discs seeming to be infertile were removed from the vitelline membrane, put into 0.9% NaCl solution and stained on a slide with propidium iodide (PI; P4170, Sigma-Aldrich Ltd, Budapest, Hungary). In case of fertile egg, propidium iodide (DNA-specific fluorescent dye of red color) stains the nucleus of the dividing embryonic cells which appear in lighting points [21].

### Statistical Analyses

For statistical analysis of the data *Mann-Whitney test* and *Kruskal-Wallis test* were used (Statistica, Version 7.0, StatSoft Hungary Ltd., Budapest, Hungary).

## Results

### In vitro Examinations

**Figure 1:**
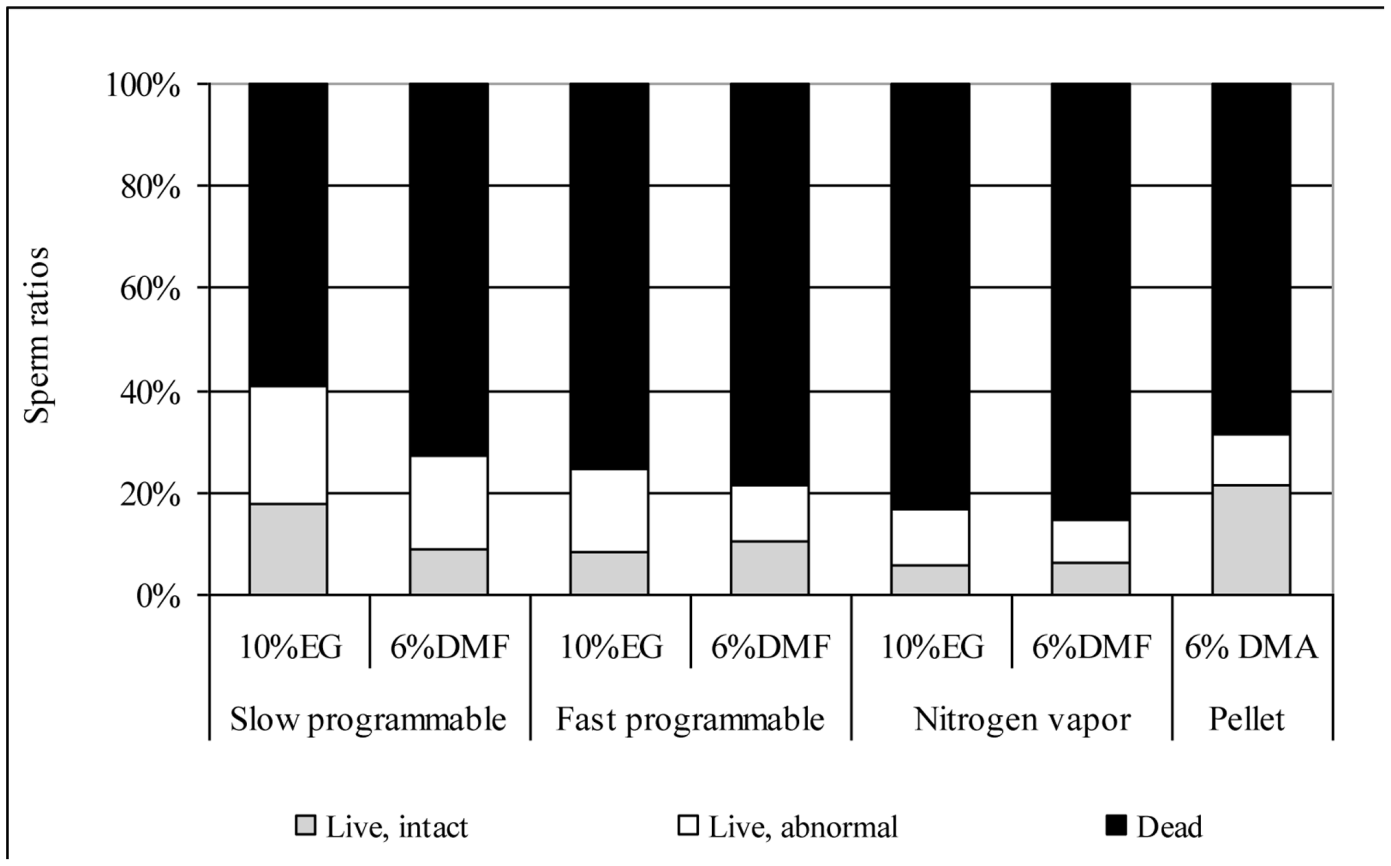
Quality of frozen/thawed spermatozoa in the different protocols.

The quality of spermatozoa after freezing and thawing is shown (Fig. 1). The best live sperm ratio (41.1%) was in the slow protocol with 10% EG; however, more than the half of these sperm were morphologically abnormal. With the same slow cooling rate using 6% DMF there were only 27.1% live sperm (p≤0.05). The same CPs with a faster cooling rate resulted in only 24.5 (for 10% EG) and 21.7% (for 6% DMF) live spermatozoa after thawing. The worst live cell ratios (16.8 and 14.8%) were in samples originating from nitrogen vapor, whereas the pellet method also resulted in high live sperm ratio (31.4%) and the highest live, intact cell ratio (21.4%).

**Figure 2:**
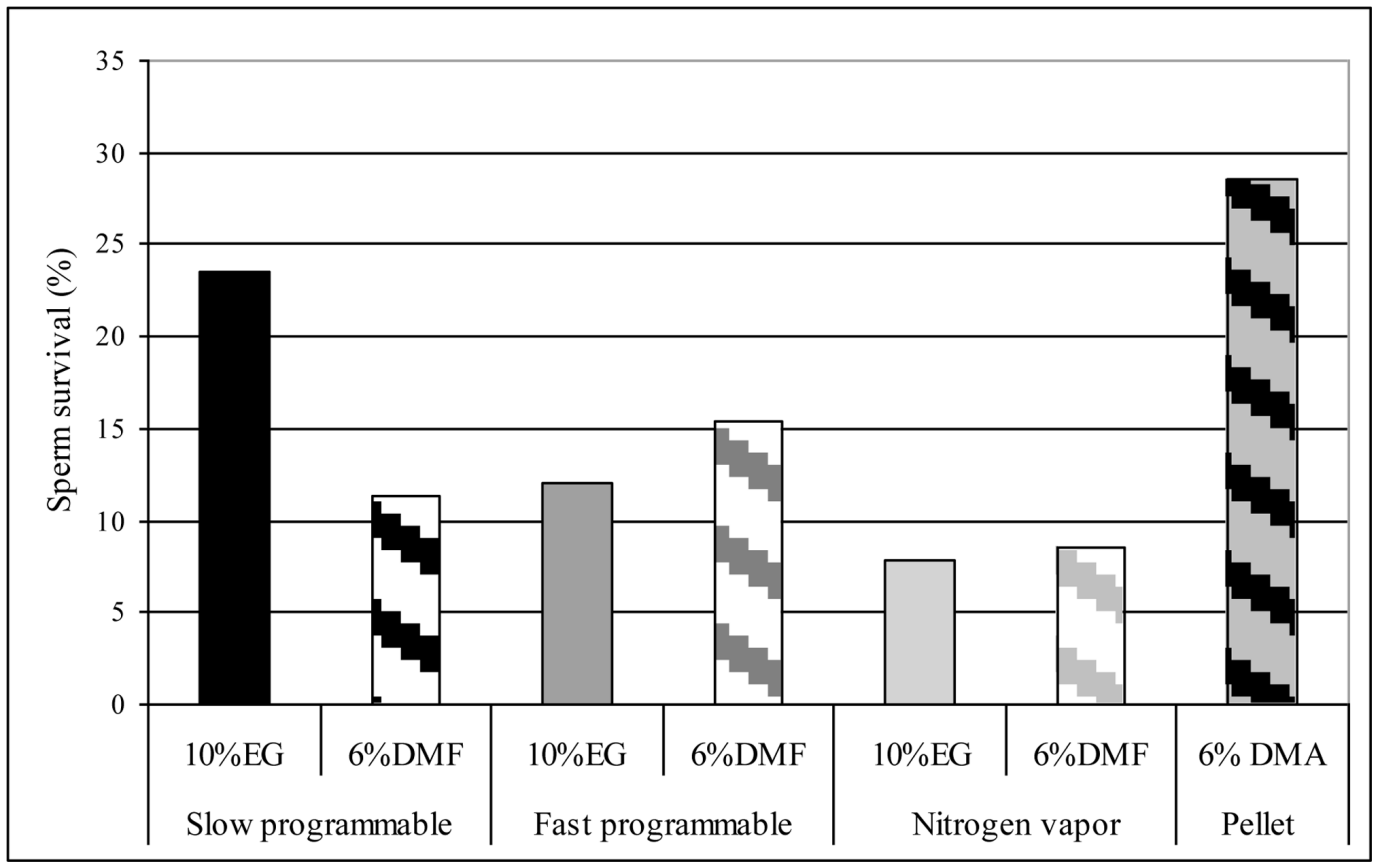
Survival rate of live, intact spermatozoa after various freezing methods.

The best (p≤0.05) survival rate of live, intact spermatozoa was in the pellet method and slow programmable protocol with 10% EG with 28.6 and 23.5%, respectively. The efficiency of the fast programmable protocol was rather low in both cases (12 and 15.5%), slow protocol with 6% DMF (11.4%) whereas freezing in nitrogen vapor produced the lowest survival rate (7.9 and 8.6%), irrespective of the type of CPs (Fig. 2).

### Artificial Insemination

**Figure 3:**
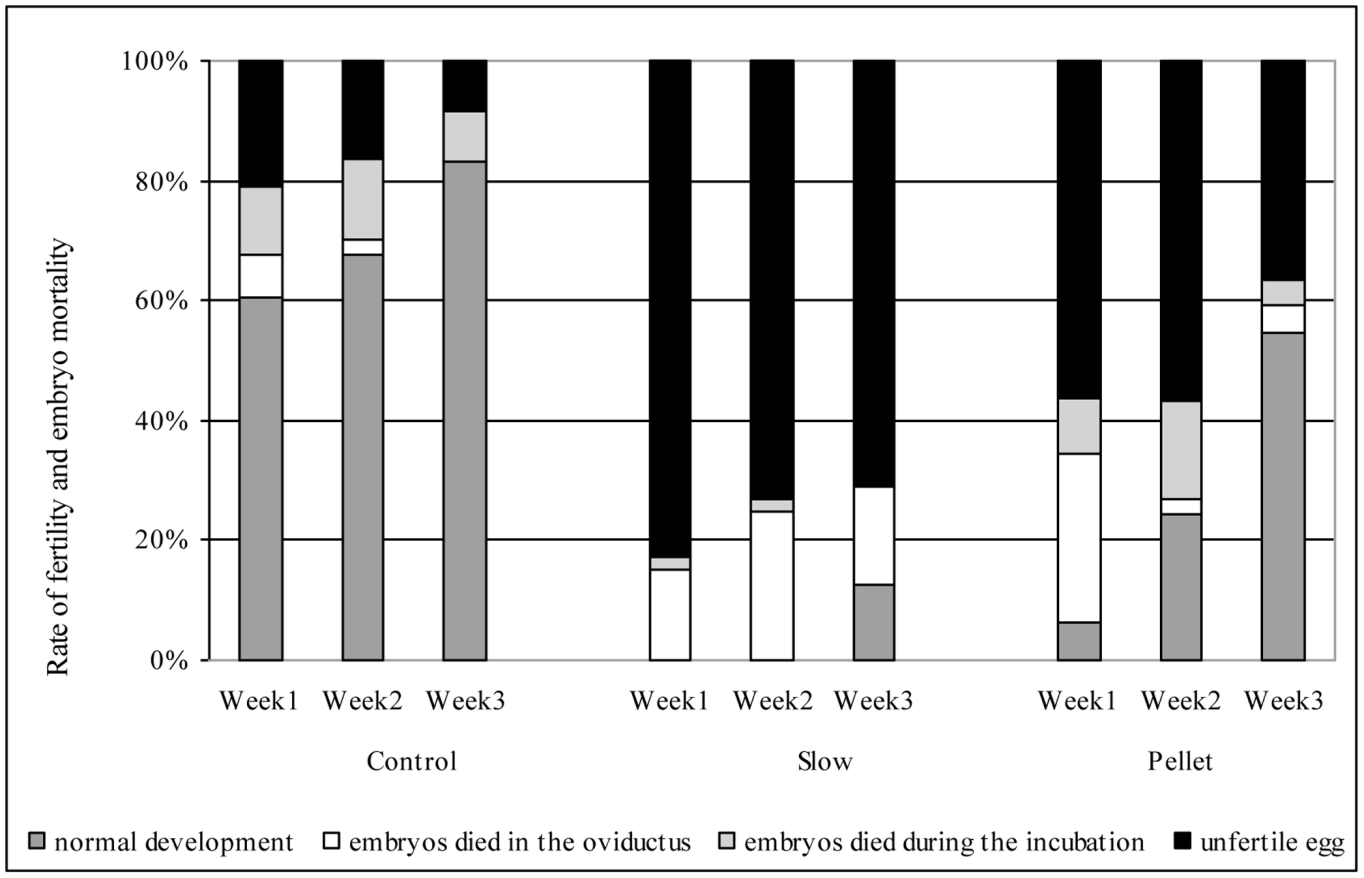
Fertility and embryo mortality in the three experimental groups following the artificial inseminations.

The slow protocol with 10% EG and pellet method yielded the best post-thaw sperm quality (based on *in vitro* assessments). Therefore, spermatozoa cryopreserved by these two methods were used for artificial insemination (*in vivo* test). To the end of the 3-week insemination period, fertility rates were 91.7, 29.1 and 63.6%, and rate of normal embryos were 83, 12, and 55% in fresh samples (control), slow and pellet method protocols, respectively. In sperm cryopreserved by the slow freezing, only dead embryos were detected in the first two weeks in the eggs. The rate of early embryonic deaths (embryo died in the oviduct) was quite high in the first week of insemination period (fresh 7, slow 15, pellet method 20%), but by the third week it had decreased significantly except for the slow protocol’ group (fresh 0, pellet method 4.5%). The rate of the embryos that died during incubation decreased in all experimental groups to the end of the examined period (Fig. 3).

## Discussion

Based on *in vitro* assessment, 6% DMF was a less effective cryoprotectant when used with a slow freezing rate, whereas 10% EG resulted in acceptable survival. It was noteworthy that the pellet method was the most promising for both sperm survival and fertility rate. This method has already been successfully adapted to turkey sperm freezing [22] with 42% sperm survival. Unfortunately, fertility was not reported. The pellet method produced higher fertility rate in the present study than that reported earlier in guinea fowl [12] using DMF with moderate freezing rate (64 vs 20%, respectively). The slow protocol with 10% EG had a good survival rate of live, intact spermatozoa, but fertility was very low. Consequently, using only *in vitro* tests to assess the effectiveness of sperm cryopreservation methods can be very misleading. Notable, there were higher rates of morphologically abnormal sperm in slower protocols than in the faster ones (nitrogen vapour or pellet method). The significantly highest rate of abnormal spermatozoa was detected in slow protocol using EG. Regarding specific defects, damage to the midpiece was the most common abnormality in all protocols (data not shown). Based on the present experiment, cryoprotectant had a lesser impact on sperm morphology than the rate of freezing. To our knowledge, there are no data available regarding sperm abnormalities of frozen thawed guinea fowl semen. However analysed the abnormal sperm ratio in frozen-thawed gander spermatozoa, the rate of midpiece injuries, namely “bent neck”, were the most prevalent [23], similar to findings with emu spermatozoa [24].

When semen was preserved by the *slow* protocol, only 18–30% of eggs were fertilized; moreover 80–90% of them contained early dead embryos. According to earlier reports frozen–thawed avian spermatozoa are associated with an increased incidence of early embryonic mortality. Although only a single spermatozoon is required to activate an ovum, in the case of avian species, polyspermic fertilization occurs and an optimal number of sperm number are necessary for good fertility. The role of the other spermatozoa penetrated into the ovum is not fully understood yet. Previous studies reported that both too low [25], [26] and high [27] sperm concentration increase the chance of embryo mortality. Presumably, in the case of the present slow protocol there were not enough live, morphologically normal spermatozoa for the complete embryo development. Since the effect of freezing protocols was compared in the study, approximately similar amounts of sperm were used for inseminations. Certainly, the higher the sperm concentration, the higher the rate of intact spermatozoa and therefore the fertility. However, it is known that there is a limit in insemination doses since significant part of sperm is rejected from the cloaca if the doses are higher than 200 µL.

Furthermore, perhaps morphologically normal spermatozoa contained cryopreservation-induced damage to nuclear structures, including DNA [28]. In humans, DNA-damaged spermatozoa fertilized ova, but the rate of early embryonic death increased [29]. During storage of equine sperm, there is also a measurable increase in DNA damage as detected by the comet assay with both cooled and frozen storage [30], [31].

In conclusion, the pellet method was the most effective way for conservation of guinea fowl spermatozoa with a high rate of fertility. There are no data available about the long term duration of sperm in pellet form. *Tselutin et al.,* [17] prefer slow freezing protocols to pellet method for special long term gene conservation purposes in the case of domestic fowl. Further investigations are in progress to check the effects of storage conditions in the case of pellet form.
